# The oxygen-independent metabolism of cyclic monoterpenes in *Castellaniella defragrans* 65Phen

**DOI:** 10.1186/1471-2180-14-164

**Published:** 2014-06-21

**Authors:** Jan Petasch, Eva-Maria Disch, Stephanie Markert, Dörte Becher, Thomas Schweder, Bruno Hüttel, Richard Reinhardt, Jens Harder

**Affiliations:** 1 Department of Microbiology, Max Planck Institute for Marine Microbiology, Celsiusstraße 1, Bremen D-28359, Germany; 2 Institute of Marine Biotechnology, Greifswald, Germany; 3 Max Planck Genome Centre Cologne, Cologne, Germany; 4 Department of Pharmaceutical Biotechnology, Ernst-Moritz-Arndt-University, Greifswald, Germany; 5 Department of Microbiology, Ernst-Moritz-Arndt-University, Greifswald, Germany

**Keywords:** Monoterpene, Isoprenoids, Biodegradation, Limonene, Phellandrene

## Abstract

**Background:**

The facultatively anaerobic betaproteobacterium *Castellaniella defragrans* 65Phen utilizes acyclic, monocyclic and bicyclic monoterpenes as sole carbon source under oxic as well as anoxic conditions. A biotransformation pathway of the acyclic *β*-myrcene required linalool dehydratase-isomerase as initial enzyme acting on the hydrocarbon. An in-frame deletion mutant did not use myrcene, but was able to grow on monocyclic monoterpenes. The genome sequence and a comparative proteome analysis together with a random transposon mutagenesis were conducted to identify genes involved in the monocyclic monoterpene metabolism. Metabolites accumulating in cultures of transposon and in-frame deletion mutants disclosed the degradation pathway.

**Results:**

*Castellaniella defragrans* 65Phen oxidizes the monocyclic monoterpene limonene at the primary methyl group forming perillyl alcohol. The genome of 3.95 Mb contained a 70 kb genome island coding for over 50 proteins involved in the monoterpene metabolism. This island showed higher homology to genes of another monoterpene-mineralizing betaproteobacterium, *Thauera terpenica* 58Eu^T^, than to genomes of the family *Alcaligenaceae*, which harbors the genus *Castellaniella*. A collection of 72 transposon mutants unable to grow on limonene contained 17 inactivated genes, with 46 mutants located in the two genes *ctmAB* (cyclic terpene metabolism). *CtmA* and *ctmB* were annotated as FAD-dependent oxidoreductases and clustered together with *ctmE*, a 2Fe-2S ferredoxin gene, and *ctmF*, coding for a NADH:ferredoxin oxidoreductase. Transposon mutants of *ctmA, B* or *E* did not grow aerobically or anaerobically on limonene, but on perillyl alcohol. The next steps in the pathway are catalyzed by the geraniol dehydrogenase GeoA and the geranial dehydrogenase GeoB, yielding perillic acid. Two transposon mutants had inactivated genes of the monoterpene ring cleavage (*mrc*) pathway. 2-Methylcitrate synthase and 2-methylcitrate dehydratase were also essential for the monoterpene metabolism but not for growth on acetate.

**Conclusions:**

The genome of *Castellaniella defragrans* 65Phen is related to other genomes of *Alcaligenaceae*, but contains a genomic island with genes of the monoterpene metabolism. C*astellaniella defragrans* 65Phen degrades limonene via a limonene dehydrogenase and the oxidation of perillyl alcohol. The initial oxidation at the primary methyl group is independent of molecular oxygen.

## Background

Monoterpenes are structurally diverse secondary metabolites of plants. The volatile branched-chain C_10_ hydrocarbons are major constituents of essential oils and are also known to attract pollinators [1]. Monoterpenes with their characteristic scent possess antimicrobial and anti-herbivore properties and are added to various foods, cosmetics or household products as flavor or antimicrobial agents [2].

Microbes use monoterpenes as carbon and energy source. The capability to transform monoterpenes is widespread among bacteria and fungi [3]. Already in the 1960s, the aerobic metabolism of several monoterpenes (limonene and *α*-pinene) was described for soil pseudomonads and the fungus *Aspergillus niger*[4]. Pseudomonads also use the acyclic citronellol [5,6] and *Pleurotus sapidus* was found to transform the bicyclic *Δ*^3^-carene [7]. Limonene may be an intermediate in the metabolism of bicyclic [8] and acyclic monoterpenes [9].

The monocyclic limonene (4-isopropenyl-1-methylcyclohexene) is one of the most common monoterpenes [10]. Several limonene biotransformation pathways with molecular oxygen as co-substrate were described for aerobic microorganisms [11,12]: (I) the oxidation at the primary methyl group to perillyl alcohol and further oxidation; (II) the epoxidation of the ring double bond and formation of a diol; (III) the ring oxidation at the C3 position forming carveol or at the C6 position forming isopiperitenol, and (IV) the epoxidation of the double bond at the isopropenyl group. All these reactions are catalyzed by cytochrome P450 monooxygenases [13]. Alternative initial reactions involve the addition of water to a double bond. The hydration of limonene to *α*-terpineol was reported for bacterial and fungal species [14]. Anoxic conditions seem to increase the transformation rate [15] and cofactor-independent hydratases were identified to catalyze the reaction [16,17]. The further degradation of *α*-terpineol via oleuropeic acid or borneol is again dependent on molecular oxygen as co-substrate [18].

A microbial monoterpene mineralization to carbon dioxide in the absence of molecular oxygen was first observed with the enrichment and isolation of denitrifying strains [19-21]. One of these strains, *Castellaniella defragrans* 65Phen isolated with *α*-phellandrene, is able to use various acyclic, monocyclic and bicyclic monoterpenes as sole carbon and energy source. Cyclic monoterpenes require a sp^2^-hybridized C1-atom as precondition for mineralization [22]. Initial metabolite studies showed the formation of geranic acid from *β*-myrcene in cell-free extracts [23]. A pathway from the acyclic monoterpene *β*-myrcene to geranic acid was identified. *β*-Myrcene is enantiospecifically hydrated to (*S*)-(+)-linalool and further isomerized to geraniol by the linalool dehydratase-isomerase (LDI) [24,25]. The NAD^+^-dependent geraniol dehydrogenase GeoA and geranial dehydrogenase GeoB oxidize geraniol to geranial and further to geranic acid (published as GeDH and GaDH) [26]. With the development of a genetic system for *C. defragrans* 65Phen, an in-frame deletion mutant with an inactivated *ldi* gene showed no growth with the acyclic *β*-myrcene, but grew like the wild type on limonene or *α*-phellandrene [27].

In this publication, we report our search for an anaerobic pathway for cyclic monoterpene degradation in *Castellaniella defragrans* 65Phen. On the basis of the genome, expressed proteins were extracted from *α*-phellandrene- and acetate-grown cultures and identified by two-dimensional gel electrophoresis coupled to MALDI-TOF-MS as well as membrane protein-enriched LC-ESI-MS/MS. A random transposon mutagenesis with a Mini-Tn5 transposon identified genes essential for the growth on cyclic monoterpenes. Metabolites formed in cultures of several genotypes were identified by GC-MS. The observations were integrated to develop a putative degradation pathway.

## Results and discussions

### The genome of *Castellaniella defragrans*

The closed genome of *Castellaniella defragrans* 65Phen has 3,952,818 bp and an overall G+C content of 68.9%. 3616 protein-coding open reading frames (ORFs), 45 transfer RNA genes and 2 ribosomal RNA operons, comprising 5S, 16S and 23S ribosomal RNA genes, were detected in the genome. *C. defragrans* 65Phen has complete sets of genes for the citrate cycle, aerobic respiration and denitrification including nitrite reductase *nirK* and both quinol-dependent nitrite oxide reductase and cytochrome C-dependent nitrite oxide reductase type *norB*. The degradative pathways matched the observed substrate utilization [21]. The lack of growth on sugars coincided with the absence of a 6-phosphofructokinase, thus the glycolysis pathway was incomplete. The biosynthesis of sugars on anabolic pathways is ensured by a fructose-1,6-bisphosphatase type I. The comparison with other genomes revealed a high similarity to related *Alcaligenaceae* species, e.g., *Bordetella pertussis* Tohama I (average nucleotide identity (ANI) 82.1%) or *Pusillimonas* sp. T7-7 (ANI 80.7%). *C. defragrans* 65Phen shares 1954 ORFs (54%) with the published genome of *Bordetella pertussis* Tohama I (Acc. no. NC_002929). In these genes the average amino acid identity was 62%.

An exception was an island of 70 kb DNA located from base 3026577 to 3096437 (Table 1). The island is flanked upstream by a transposable element and downstream by a cluster for the degradation of amino acids. The majority of genes in the island are most similar to betaproteobacterial genes outside the *Alcaligenaceae*. The island includes the genes for the initial myrcene transformation, *ldi* for the linalool dehydratase-isomerase, *geoA* for the geraniol dehydrogenase and *geoB* for the geranial dehydrogenase*,* and the previously published contig derived from fosmids (Acc. no. FR669447). The predicted proteins (Tab. 1) resemble proteins of the monoterpene-mineralizing strains, *Thauera terpenica* 58Eu^T^[20] and *Pseudomonas* sp. 19-rlim [28], as well as of *Azoarcus* strains, which have not been tested for monoterpene degradation. *Thauera* and *Azoarcus* are *Rhodocyclales* with a well-established capacity to mineralize aromatic hydrocarbons [29]. *Pseudomonas* sp. 19-rlim belongs to the gammaproteobacterial *Pseudomonadaceae* which degrade a wide range of hydrophobic substances [30]. Many predicted proteins in the island were annotated as beta-oxidation pathway-related enzymes and as a transporter of hydrophobic substances.

**Table 1:** **Genes of the genome island and assigned functions in the metabolism of monoterpenes in *Castellaniella defragrans *** **65Phen**

**Protein_id**	**Proteine detection method**^**a**^	**2D fold change/LC enrichment level**^**b**^	**Gene annotation**	**Related gene product**			
				**E value**	**% identity**	**Organism**	**Accession no.**
CDM25240	n.d.	n.d.	Hypothetical protein	4E-60	59	*Thauera terpenica* 58Eu	EPZ16239
CDM25241	LC	++	Acyl-CoA dehydrogenase protein	0.0	82	*Thauera terpenica* 58Eu	EPZ16240
CDM25242	LC	0	Molybdopterin-binding OR	3E-70	74	*Thauera terpenica* 58Eu	EPZ16227
CDM25243	n.d.	n.d.	Tyrosine/serine phosphatase	1E-58	48	*Thauera linaloolentis* 47Lol	ENO83508
CDM25244	LC	0	2,4-dienoyl-CoA reductase	0.0	87	*Thauera terpenica* 58Eu	EPZ16243
CDM25245	n.d.	n.d.	NADH:flavin oxidoreductase	1E-179	68	*Thauera terpenica* 58Eu	EPZ16244
CDM25246	2D/LC	3.0/++	3-hydroxyacyl-CoA dehydrogenase	5E-167	82	*Azoarcus* sp. KH32C	YP_007598290
CDM25247	n.d.	n.d.	IS4 family transposase	0.0	63	*Thiomonas* sp. FB-6	WP_018915433
CDM25248, MrcH	n.d.	n.d.	MaoC-like dehydratase	2E-36	62	*Azoarcus* sp. KH32C	YP_007598291
CDM25249, MrcG	n.d.	n.d.	MaoC-like dehydratase	6E-50	67	*Thauera terpenica* 58Eu	EPZ16257
CDM25250, MrcF	LC	++	Perillyl-CoA hydratase	0.0	58	*Thauera terpenica* 58Eu	EPZ16258
CDM25251, MrcE	2D/LC	18/++	4-isopropenyl-2-oxo-cyclohexane-1-carboxyl-CoA hydrolase	4E-167	88	*Azoarcus* sp. KH32C	YP_007598294
CDM25252, MrcD	2D/LC	3.0/++	2-hydroxy-4-isopropenyl-cyclohexane-1-carboxyl-CoA dehydrogenase	1E-153	85	*Azoarcus* sp. KH32C	YP_007598295
CDM25253, MrcC	2D	2,3	2,4-dienoyl reductase	0.0	86	*Thauera terpenica* 58Eu	EPZ16261
CDM25254, MrcB	LC	++	Acyl-CoA dehydrogenase	0.0	90	*Azoarcus toluclasticus*	WP_018990727
CDM25255, MrcA	LC	++	Oxidoreductase, FAD-binding	0.0	73	*Azoarcus toluclasticus*	WP_018990723
CDM25256	2D/LC	19/++	(*R*)-specific enoyl-CoA hydratase	1E-88	85	*Thauera terpenica* 58Eu	EPZ15051
CDM25257	LC	0	Citrate lyase	6E-141	73	*Thauera terpenica* 58Eu	EPZ15052
CDM25258	LC	++	Acyl-CoA dehydrogenase	0.0	92	*Thauera terpenica* 58Eu	EPZ15053
CDM25259	LC	++	RND efflux transporter	0.0	62	*Thauera terpenica* 58Eu	EPZ15054
CDM25260	LC	++	RND efflux transporter	0.0	80	*Thauera terpenica* 58Eu	EPZ15055
CDM25261	LC	++	RND efflux transporter	4E-142	68	*Thauera terpenica* 58Eu	EPZ15056
CDM25262	LC	++	RND efflux transporter	0.0	81	*Thauera terpenica* 58Eu	EPZ15057
CDM25263	LC	++	Acetoacetyl-CoA synthetase	0.0	84	*Thauera terpenica* 58Eu	EPZ15058
CDM25264	n.d.	n.d.	Enoyl-CoA hydratase	9E-136	76	*Thauera terpenica* 58Eu	EPZ15059
CDM25265, GeoC	LC	++	Perillate--CoA ligase	0.0	71	*Thauera terpenica* 58Eu	EPZ15060
CDM25266	LC	0	Hypothetical protein	4,3	52	*Fusarium graminearum* PH-1	XP_382023
CDM25267, GeoA	2D/LC	42/++	Geraniol dehydrogenase	0.0	84	*Thauera terpenica* 58Eu	EPZ14350
CDM25268	LC	++	Hypothetical protein	8E-117	74	*Thauera terpenica* 58Eu	EPZ14349
CDM25269	n.d.	n.d.	Hypothetical protein	2E-97	69	*Thauera terpenica* 58Eu	EPZ14348
CDM25270	n.d.	n.d.	Hypothetical protein	6E-32	66	*Thauera terpenica* 58Eu	EPZ14347
CDM25271	n.d.	n.d.	Thioesterase	2E-33	46	*Magnetospirillum magneticum*	YP_420191
CDM25272, LDI	LC	++	LDI precursor protein	8E-14	25	*Stereum hirsutum* FP-91666 SS1	EIM80109
CDM25273	n.d.	n.d.	Hypothetical protein	5E-85	43	*Gordonia paraffinivorans*	WP_006900876
CDM25274	n.d.	n.d.	Hypothetical protein	5E-07	30	*Gordonia paraffinivorans*	WP_006900845
CDM25275	LC	++	Acyl-CoA dehydrogenase	4E-106	54	*Azoarcus toluclasticus*	WP_018990670
CDM25276	n.d.	n.d.	Hypothetical protein	4E-40	40	*Glaciecola punicea*	WP_006005307
CDM25277	n.d.	n.d.	Hypothetical protein	5E-21	58	*Pseudomonas* sp. 19-rlim	AEO27370
CDM25278	n.d.	n.d.	Hypothetical protein	1E-69	62	*Pseudomonas* sp. 19-rlim	AEO27371
CDM25279	LC	++	Hypothetical protein	1E-120	68	*Pseudomonas* sp. 19-rlim	AEO27372
CDM25280	n.d.	n.d.	MarR transcriptional regulator	7E-81	83	*Thauera terpenica* 58Eu	EPZ16291
CDM25281, GeoB	2D/LC	15/++	Geranial dehydrogenase	0.0	91	*Thauera terpenica* 58Eu	EPZ16290
CDM25282	LC	++	Acyl-CoA dehydrogenase	0.0	89	*Thauera terpenica* 58Eu	EPZ16289
CDM25283	n.d.	n.d.	LuxR family transcriptional regulator	0.0	59	*Thauera terpenica* 58Eu	EPZ16271
CDM25284, CtmG	LC	++	Hypothetical protein	5E-35	39	*Azoarcus* sp. KH32C	YP_007598506
CDM25285, CtmF	LC	++	NADH:ferredoxin oxidoreductase	4E-147	56	*Caulobacter* sp*.* AP07	WP_007674692
CDM25286, CtmE	2D	6.3/++	Ferredoxin, 2Fe-2S	1E-32	50	*Caulobacter crescentus* CB15	NP_422318
CDM25287, CtmD	n.d.	n.d.	Hypothetical protein	4,1	38	*Ochrobactrum* sp. CDB2	WP_007881652
CDM25288, CtmC	n.d.	n.d.	Hypothetical protein	2,3	33	*Bombus impatiens*	XP_003489707
CDM25289, CtmB	2D/LC	3.4/++	Limonene dehydrogenase	4E-131	41	Deltaproteobacterium NaphS2	WP_006422074
CDM25290, CtmA	LC	++	Limonene dehydrogenase	5E-57	30	Deltaproteobacterium NaphS2	WP_006422074
CDM25291	LC	++	Acetyl-CoA acetyltransferase	0.0	79	*Thauera terpenica* 58Eu	EPZ16237
CDM25292	LC	0	MarR transcriptional regulator	2E-76	77	*Thauera terpenica* 58Eu	EPZ16235
CDM25293	n.d.	n.d.	Hypothetical protein	4E-24	56	*Thauera terpenica* 58Eu	EPZ16283
CDM25294	LC	++	Hypothetical protein	2E-83	68	*Thauera terpenica* 58Eu	EPZ16282
CDM25295	n.d.	n.d.	Hypothetical protein	6E-155	76	*Thauera terpenica* 58Eu	EPZ16281
CDM25296	n.d.	n.d.	MarR transcriptional regulator	5E-94	81	*Thauera terpenica* 58Eu	EPZ16280
CDM25297	n.d.	n.d.	Hypothetical protein	2E-168	72	*Thauera terpenica* 58Eu	EPZ16232
CDM25298	LC	++	Hypothetical protein	0.0	72	*Thauera terpenica* 58Eu	EPZ16231
CDM25299	LC	++	Acetyl-CoA acetyltransferase	0.0	90	*Thauera terpenica* 58Eu	EPZ16230
CDM25300	n.d.	n.d.	Acetyl-CoA hydrolase/transferase	0.0	74	*Thauera terpenica* 58Eu	EPZ16229
CDM25301	LC	++	Electron transfer flavoprotein	7E-133	78	*Thauera terpenica* 58Eu	EPZ16226
CDM25302	n.d.	n.d.	Electron transfer flavoprotein	7E-160	76	*Azoarcus* sp. KH32C	YP_007552025

^a^2D, identified with 2D-SDS-PAGE and MALDI-TOF-MS; LC, identified with LC-ESI-MS/MS.

^b^++ peptides only identified in α-phellandrene fraction, + increase in α-phellandrene fraction, 0 ratio remained unchanged.

### The proteome of monoterpene utilization

The soluble protein fractions of bacteria grown on acetate or on α-phellandrene were analyzed by two-dimensional gel electrophoresis, followed by enzymatic digest and MALDI-TOF mass spectrometry. The enriched membrane protein fractions were analyzed by one-dimensional gel electrophoresis, enzymatic digest and LC-ESI-MS/MS. 234 and 851 individual proteins of *C. defragrans* 65Phen were identified with 2D-SDS-PAGE separation and MALDI-TOF-MS, and with LC-ESI-MS/MS, respectively. The monoterpene proteome, defined as proteins induced in extracts of α-phellandrene-grown cells in comparison to extracts of acetate-grown cells, included a total of 107 proteins, of which 28 proteins were identified by MALDI-TOF-MS and 97 proteins were identified by LC-ESI-MS/MS, with an overlap of 18 proteins that were identified by both techniques (Additional file 1: Table S1). 32 of these proteins are encoded by genes in the island including the enzymes LDI, GeoA and GeoB. Among the 75 α-phellandrene-induced proteins with a gene location outside of the island, ABC transporter-associated proteins were highly up-regulated.

### Growth on oxidized limonene metabolites

**Figure 1:**
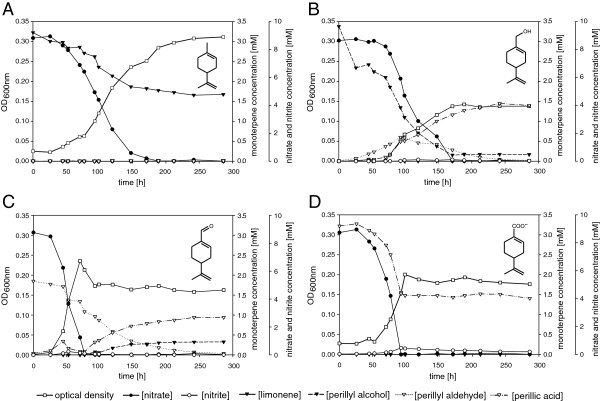
**Anaerobic growth of *C. defragrans *65Phen on limonene and putative metabolites.** Limonene **(A)**, perillyl alcohol **(B)**, perillyl aldehyde **(C)** and perillic acid **(D)** were tested as growth substrate, each with a concentration of 3 mM and nitrate limitation (10 mM).

The geraniol dehydrogenase GeoA is an allyl-alcohol dehydrogenase with the highest catalytic activity on perillyl alcohol [26]. The high expression of GeoA and GeoB in α-phellandrene-grown cells questioned the utilization of the cyclic monoterpene alcohol by *C. defragrans*. The strain 65Phen grew on perillyl alcohol, perillyl aldehyde and perillic acid (Figure 1B-D). During active denitrification, perillyl aldehyde accumulated transiently in the culture growing on perillyl alcohol (Figure 1B). Perillic acid accumulated in cultures growing on the alcohol and the aldehyde. When the electron acceptor nitrate was depleted, the cells disproportionated perillyl aldehyde into perillyl alcohol and perillyl acid (Figure 1B, C). During growth on limonene (Figure 1A), the perillyl derivatives were not detected suggesting a rate-limitation in the pathway by the initial limonene-transforming enzyme.

### Transposon insertion mutagenesis

To identify the genes involved in monoterpene degradation, a random insertion mutagenesis with a mini-Tn5 transposon was performed according to Larsen *et al*. [31]. Insertion mutants of *C. defragrans* 65Phen were obtained on agar plates with a minimal medium and acetate and nitrate as carbon source and electron acceptor, respectively, and rifampicin and kanamycin as selective compounds. These colonies were transferred to acetate-free anoxic plates with nitrate and supplied with limonene via the gas phase. Six of 1000 insertion mutants revealed a lack of growth under these conditions. Our collection of 72 mutant strains covered 46 unique insertion positions in 17 genes. The majority of insertions yielding a loss of function were in *ctmA*. It was inactivated in 45 mutants at 22 different positions. The adjacent gene *ctmB* was inactivated thrice at different positions. Also a gene closely located, *ctmE,* was inactivated at two insertion sites. Five genes were present at least twice in the mutant collection, whereas nine other genes were present once. The five genes coded CDM25082, CDM25239, CDM25260, CDM25338 and CDM25923.

Anaerobic denitrifying growth of selected transposon mutants was tested in liquid culture (Table 2). All transposon mutants denitrified and grew with acetate in liquid culture. In liquid culture on limonene, four strains, the mutants of a putative transcriptional regulator (CDM25239), a putative inner membrane protein (CDM25338), a hypothetical protein (CDM25510) and a plasmid stability protein (CDM24643) grew similarly to the wild type in liquid culture. No colony formation on plates, but growth in liquid medium coincided with a reduced mass transfer limitation of limonene in liquid culture. Three mutant strains showed weak growth and ten mutant strains did not grow on limonene in liquid culture (Table 2). Additional growth experiments showed that the genes *ctmA*, *ctmB* or *ctmE* were not required for growth on perillyl alcohol. This physiology was also observed in aerobic cultures growing in the absence of nitrate (Additional file 2: Figure S1). The three genes are part of an operon-like cluster which was named *ctmABCDEFG*, for cyclic terpene metabolism-associated genes (Table 1). Also other monoterpenes tested (Table 2) did not support growth of these three mutants.

**Table 2:** **Growth of *Castellaniella defragrans *** **65Phen transposon insertion mutants in liquid medium**

**Inactivated gene**	**Annotation**	**Length [b]**	**Insertion positions [b]**	Growth substrate^*^			
				**Acetate**	**Limonene**	***β*-Myrcene**	**Further substrates**
Initial oxidation							
CDM25290 CtmA	Limonene dehydrogenase, alpha subunit	1698	133	+	-	-	Perillyl alcohol (+), α-phellandrene (-), α-pinene (-), β-pinene (-)
CDM25290 CtmA	Limonene dehydrogenase, alpha subunit	1698	1188	+	-	-	Perillyl alcohol (+), α-phellandrene (-), α-pinene (-), β-pinene (-)
CDM25289 CtmB	Limonene dehydrogenase, beta subunit	1650	38	+	-	-	Perillyl alcohol (+), α-phellandrene (-), α-pinene (-), β-pinene (-)
CDM25289 CtmB	Limonene dehydrogenase, beta subunit	1650	559	+	-	-	Perillyl alcohol (+), α-phellandrene (-), α-pinene (-), β-pinene (-)
CDM25286 CtmE	Ferredoxin, 2Fe-2S	324	111	+	-	-	Perillyl alcohol (+), α-phellandrene (-), α-pinene (-), β-pinene (-)
Ring cleavage and β-oxidation							
CDM23589	Electron transfer flavoprotein:ubiquinone oxidoreductase	1647	138	+	(+)	(+)	Perillic acid ((+))
CDM25250 MrcF	Perillyl-CoA hydratase	1239	18	+	-	-	α-phellandrene (-)
CDM25253 MrcC	2,4-dienoyl-CoA reductase	888	841	+	-	-	α-phellandrene (-)
CDM25297	Hypothetical protein	1023	34	+	-	-	
CDM258488	Electron transfer protein	888	385	+	-	-	Perillic acid (-)
CDM25923	Enoyl-CoA hydratase	777	461	+	-	-	
Methylcitrate cycle							
CDM25082	2-methylcitrate dehydratase	1452	262	+	-	-	
CDM25081	2-methylcitrate synthase	1239	-18	+	(+)	+	
Other functions							
CDM23676	Molybdenum transport system protein	865	47	+	-	-	Perillyl aldehyde (-)
CDM25260	RND efflux transporter, periplasmic component	1356	24	+	(+)	(+)	Perillic acid ((+))

* + growth like wild type; - no growth; (+) decreased growth rate and maximum density compared to wild type.

### Metabolite formation from limonene

**Figure 2:**
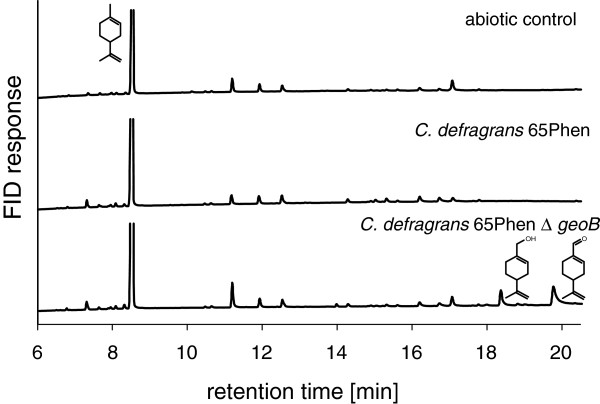
**GC chromatogram of metabolite accumulation.** Extracts of nitrate-limited cultures were obtained from the primary stationary phase of cultures grown with 20 mM acetate as co-substrate and 3 mM (*R*)-(+)-limonene.

To demonstrate the *in vivo* formation of perillyl alcohol from limonene, an in-frame deletion mutant of *geoB* was generated. The deletion of the alcohol dehydrogenase gene *geoA* caused a decreased growth rate and biomass formation with several monoterpenes as growth substance. We attribute this residual growth with the expression of a second alcohol dehydrogenase in cells of the in-frame *ΔgeoA* mutant [27]. The deletion mutant *C. defragrans* 65Phen *ΔgeoB* grew on acetate and perillic acid, but lacked the ability to grow on limonene, *β*-myrcene, perillyl alcohol and perillyl aldehyde (data not shown). To analyze the metabolite formation, the mutant and for comparison the wild type were anaerobically cultured with 3 mM (*R*)-(+)-limonene and 20 mM acetate as co-substrates in the presence of 20 mM nitrate. To increase the mass transfer, the organic carrier phase 2,2,4,4,6,8,8-heptamethylnonane (HMN) - and with it the two-phase system - was replaced by 0.5% v/v Tween 20 in a homogeneous phase. In the early stationary phase after seven days, hydrophobic compounds were salted out and extracted with isopropanol. The metabolites in the isopropanol phase were analyzed stereospecifically by GC and GC-MS (Figure 2). (*R*)-(+)-perillyl alcohol as well as (*R*)-(+)-perillyl aldehyde were not detected in an abiotic control without cells and in the culture of the wild type, but in the deletion mutant *ΔgeoB*. This *in vivo* formation of (*R*)-(+)-perillyl alcohol from (*R*)-(+)-limonene suggested an oxidation of limonene at the methyl group as initial reaction of the degradation pathway.

### Hypothetical pathway of limonene degradation based on genome, monoterpene proteome and the physiology of transposon mutants

The genome island of *C. defragrans* 65Phen is related to genes of *T. terpenica* 58Eu^T^ which also mineralizes cyclic monoterpenes anaerobically [21]. A related gene cluster from *Pseudomonas* sp. strain 19-rlim has been deposited, but not described (Genbank JN379031). Of these strains, only *C. defragrans* 65Phen has the cyclic terpene metabolism cluster that contained two FAD-dependent oxidoreductases (*ctmA* and *ctmB*), a 2Fe-2S ferredoxin (*ctmE*) and a ferredoxin reductase (*ctmF*) together with three hypothetical genes (*ctmCD* and *ctmG*) and a putative transcriptional regulator of the luxR family. CtmA, B, E and F were up-regulated proteins in *α*-phellandrene-grown cells, in comparison to acetate-grown cells. The other proteins of the cluster were not detected. The physiology of the transposon mutants in *ctmA, ctmB* and *ctmE* together with the formation of perillyl alcohol in cultures of the *ΔgeoB* mutant suggests that the Ctm subunits represent a novel enzyme for hydrocarbon activation, catalyzing the oxidation of a methyl group from limonene to the corresponding alcohol. More precisely, a methyl group of an allyl group is oxidized, as it was demonstrated that the carbon-carbon double bond is required for the metabolism [22]. So far, the activation of hydrocarbons in the absence of molecular oxygen is known to be catalyzed by glycine radical enzymes (alkanes, toluene) or molybdenum-containing enzymes (ethylbenzene, cholesterol) [29]. The Ctm enzyme seems to comprise a catalytic core of CtmAB and an electron transfer chain consisting of CtmEF. CtmAB are both annotated as FAD-dependent oxidoreductases, but have a low amino acid identity to each other (27%). The domain structure of both oxidoreductases is similar to those of phytoene dehydrogenases (COG1233, E value of 1e^-60^) which introduce symmetrically double bonds at phytoene adjacent to existing carbon-carbon double bonds. This process is also an oxidation of the alkyl part of an allylic group. Oxidized dinucleotides like FAD were described as electron acceptors for bacterial phytoene dehydrogenases [32]. Among the COG1233 enzymes, CtmAB showed the largest gene identity to a putative oxidoreductase of the deltaproteobacterial obligate anaerobic sulfate-reducing strain NaphS2 [33]. In contrast, *ctmEF* is phylogenetically related to genes of the alphabacterial *Caulobacter* species. Ferredoxins and ferredoxin reductases are well known as electron transfer chain from the NADH/NADPH-pool to cytochrome P450 monooxygenases in aerobic bacteria [13]. Transposon mutants of *ctmA, ctmB* or *ctmE* lacked the capability to mineralize the acyclic *β*-myrcene or the bicyclic *α*-pinene. Thus, enzymes of the *ctm* cluster may also be involved in the metabolism of these monoterpenes in *C. defragrans* 65Phen.

The oxidation of perillyl alcohol involved GeoA, previously identified as geraniol dehydrogenase with a broad substrate spectrum, and GeoB, previously identified as geranial dehydrogenase [26]. The in-frame deletion of *geoB* revealed *in vivo* the co-metabolic transformation of limonene to perillyl alcohol and further to perillyl aldehyde. Both GeoA and GeoB were highly expressed in cells grown on *α*-phellandrene. Another aldehyde dehydrogenase gene (CDM24151) with unknown substrate specificity was also expressed in cells grown on *α*-phellandrene, but the level of induction was lower than that of GeoB. The gene was located outside the monoterpene island and was related to an aldehyde dehydrogenase of *Pusillimonas* sp. T7-7 (84% amino acid identity). The activation of perillic acid to a coenzyme A thioester may be catalyzed by the induced CDM26265, an ATP-dependent ligase that we annotated as GeoC due to the gene location next but one near *geoA*.

**Figure 3:**
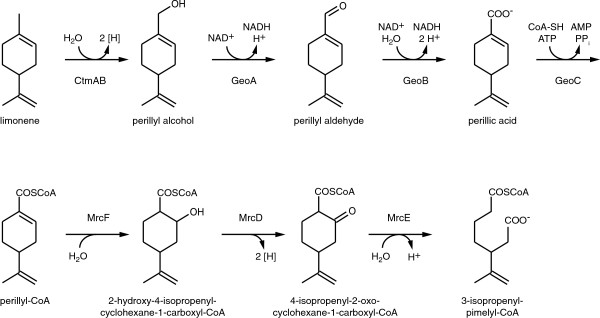
**Proposed partial degradation pathway of monocyclic limonene by *C. defragrans *65Phen*.*** Enzymes of *C. defragrans* 65Phen predicted to catalyze reactions of the limonene metabolism: CtmAB, limonene dehydrogenase; GeoA, geraniol dehydrogenase; GeoB, geranial dehydrogenase; GeoC perillate-CoA ligase; MrcF, perillyl-CoA hydratase; MrcD, 2-hydroxy-4-isopropenylcyclohexane-1-carboxyl-CoA dehydrogenase; MrcE, 4-isopropenyl-2-oxocyclohexane-1-carboxyl-CoA hydrolase (Ctm cyclic terpene metabolism, Mrc monoterpene ring cleavage).

The ring cleavage of the cyclic perillyl-CoA resembles the cyclohex-1-ene-1-carboxyl-CoA degradation in anaerobic benzoate degraders or the described monocyclic monoterpene degradation pathways of *Pseudomonas putida*[34] or *Geobacillus* (*ex. Bacillus*) *stearothermophilus*[35] (Figure 3). We named the genes monoterpene ring cleavage-associated genes (*mrc*). Perillyl-CoA may be hydrated by MrcF to 2-hydroxy-4-isopropenylcyclohexane-1-carboxyl-CoA. Oxidation by the dehydrogenase MrcD may yield 4-isopropenyl-2-oxocyclohexane-1-carboxyl-CoA that may be hydrolysed by MrcE to 4-isopropenylpimelyl-CoA. Related enzymes of the anaerobic benzoate catabolism, BadK, BadH and BadI, catalyze the β-oxidation-like oxidation of cyclohexenecarboxyl-CoA, forming pimelyl-CoA. Among the strains of *Azoarcus* and *Thauera*, the enzymes of *Azoarcus* sp. KH32C are most closely related to MrcDEF. BadK (YP_007598293) catalyzes the hydration of cyclohex-1-ene-1-carboxyl-CoA and has an amino acid identity of 53% to MrcF, affiliating to the enoyl-CoA hydratase/isomerase superfamily. The dehydrogenase BadH (YP_007598295) has a high identity of 85% to MrcD. The formation of pimelyl-CoA is catalzyed by BadI (YP_007598294) which is highly similar to MrcE (88% identity). All three proteins MrcDEF were expressed in cells grown on *α*-phellandrene. In *C. defragrans* 65Phen, the genes required for ring cleavage are located in a cluster (*mrcABCDEFGH*). An insertion mutant of *mrcF* did not grow on monoterpenes like limonene or *α*-phellandrene. A second mutant strain with an insertion in the gene *mrcC* also lacked the capability to grow on limonene. *MrcC* was annotated as a 2,4-dienoyl reductase.

The following degradation of 4-isopropenylpimelyl-CoA may include 3 steps of β-oxidation-like degradation forming three acetyl-CoA and methacrylyl-CoA which may be decarboxylated in a valine-like degradation to propanoyl-CoA. Propanoyl-CoA is connected to the TCA cycle via the methylcitrate cycle. Several enzymes of the valine degradation were up-regulated, but enzymes of the methylcitrate cycle were detected without expression differences between the substrate conditions. However, transposon mutants in 2-methylcitrate synthase and 2-methylcitrate dehydratase showed impaired growth on limonene. Genes assigned to the valine degradation and methylcitrate cycle were located outside of the genome island.

The complete genome of *C. defragrans* did not show the pathway for the utilization of acyclic monoterpenes as defined by the studies on citronellol in pseudomonads [5]. The *atu* and *liu* genes encode two pathways of enzymes required to transform geranyl-CoA to acetyl-CoA and acetoacetate. The *liu* genes representing the leucine degradation via 3-methylbut-2-enoyl-CoA were present in the genome of *C. defragrans* 65Phen, but the genes for the acyclic terpene utilization (*atu*) were not identified. Atu enzymes catalyze the carbon chain cleavage from an acyclic ten-carbon carboxyl-CoA ester to 3-methyl-crotonyl-CoA which is further degraded in the *liu* pathway to acetyl-CoA and acetoacetate. The tertiary carbon atom undergoes a carbon-carbon cleavage after carboxylation of the methyl group by AtuC/AtuF. Homologues to these pathway-specific genes *atuC*/*atuF* were not present in the genome of *C. defragrans*. The absence of *atu* genes and the lack of growth on myrcene exhibited by transposon mutants of the limonene degradation pathway is first evidence for a connection between mycrene and limonene metabolism. Other initial reports on the biological formation of cyclic monoterpenes from myrcene [9] and linalool [36] are known, but the molecular basis for this reactions have not been revealed so far.

## Conclusions

The degradation of monocyclic monoterpenes in *C. defragrans* 65Phen is initiated at the methyl group via oxidation by a new enzyme belonging to the phytoene dehydrogenase family (Cog1233). Ferredoxin and ferredoxin reductase are involved in the oxidation of limonene to perillyl alcohol. Expressed proteins and transposon mutants indicate that the further degradation pathway is an oxidation to perillic acid, followed by activation to perillyl CoA thioester and a ring cleavage. Most of the genes for these pathways are located on a genetic island, named the monoterpene island, and seemed to be acquired by horizontal gene transfer from aerobic as well as from anaerobic bacteria.

## Methods

### Bacterial strains, plasmids and culture conditions

*C. defragrans* 65Phen-RIF, a strain containing a rifampicin resistance, was used in this study in synonym for *C. defragrans* 65Phen wild type [27]. Strains of *C. defragrans* and *E. coli* as well as plasmids used in this work are listed in Table 3. Additional transposon insertion mutants used in this study are listed in Table 2.

**Table 3:** Bacterial strains and plasmids used in this work

**Strain or plasmid**	**Relevant characteristics**	**Source or reference**
Strains		
*C. defragrans*		[21]
65Phen-RIF	Ra^R^	[27]
*ΔgeoB*	65Phen-RIF, Ra^R^, *ΔgeoB*	This study
*ctmA*::Tn5a	65Phen-RIF, Ra^R^, Km^R^, transposon insertion in *ctmA* at position 133	This study
*ctmA*::Tn5b	65Phen-RIF, Ra^R^, Km^R^, transposon insertion in *ctmA* at position 1188	This study
*ctmB*::Tn5a	65Phen-RIF, Ra^R^, Km^R^, transposon insertion in *ctmB* at position 38	This study
*ctmB*::Tn5b	65Phen-RIF, Ra^R^, Km^R^, transposon insertion in *ctmB* at position 559	This study
*ctmE*::Tn5	65Phen-RIF, Ra^R^, Km^R^, transposon insertion in *ctmE* at position 111	This study
*E. coli*		
W20767	RP4-2-*tet*::Mu-1, *kan*::Tn7 integrant, *leu-63*::IS*10, recA1, creC510, hsdR17, endA1, zbf-5, uidA, (ΔMluI)::pir + thi*	[37]
S17-1	*Thi*, *pro*, *hsdR*, *recA* with RP4-2[Tc::Mu-Km::Tn7]	[38]
Plasmids		
pRL27	Tn5 with KmR, R6K *ori*, *oriT*, RP4, *tnp*	[31]
pCR4-TOPO	Am^R^, Km^R^, *lacZα*	Invitrogen
pK19mobsacB	Km^R^, *mob*, *sacB* modified from *B. subtilis*, *lacZα*	[39]
pK19mobsacB*ΔgeoB*	Km^R^, *mob*, *sacB* modified from *B. subtilis*, *lacZα*, 2000b flanking regions of *ΔgeoB*	This study

Preparation of anoxic mineral media and anaerobic cultivation was performed as previously described with small modifications [21]. Media were buffered with 10 mM NaH_2_PO_4_ pH 7.2, vitamins were not added and the headspace consisted of N_2_ gas. Growth experiments were performed in 10 mL media and 300 μL HMN. Inocula were 2% (v/v) of a freshly grown culture. Cultures were incubated at 28°C and shaken at 90 rpm. The optical density was measured directly at 660 nm. Monoterpenes used in this study were purchased from Sigma-Aldrich (Taufkirchen, Germany) with 95 to 97% purity.

### Metabolite analysis

For metabolite analysis, triplicates of *C. defragrans* 65Phen cultures and non-inoculated controls were grown in 500 mL culture flask with 400 mL medium. The mineral medium was autoclaved in the flasks and the headspace was replaced with nitrogen immediately afterwards. After cooling to 21°C, trace minerals, 20 mL HMN, 3 mM monoterpenes (limonene, perillyl alcohol, perillyl aldehyde or perillic acid) and 0.5% inoculum were added. For each measurement 50 μL of the HMN phase and 1 mL of the aqueous phase were sampled. In total, a maximum of 5% (v/v) of each phase was sampled. 1 μL of the organic phase was analyzed by gas chromatography with flame ionization detection (PerkinElmer Auto System XL, Überlingen, Germany). Separation was performed on an Optima-5 column (50 m × 0.32 mm, 0.25 μm film thickness; Macherey-Nagel, Düren, Germany) with the following temperature program: injection port temperature 250°C, detection temperature 350°C, initial column temperature 60°C for 3 min, increasing to 120°C with a rate at 3°C min^-1^, staying constant for 0.1 min, further increasing to 320°C at 40°C min^-1^ and hold for 3 min. The split ratio was set to 1:8. All concentrations refer to the aqueous phase. The sample of the aqueous phase was analyzed for optical density at 600 nm and for nitrate and nitrite concentrations as described [19]. Organic acids were separated on a reverse phase HPLC with a Nucleodur C18 Isis column (25 cm × 4.6 mm, 5 μm spheres; Macherey-Nagel, Düren, Germany). The mobile phase consisted of 36% (v/v) acetonitrile and 64% (v/v) 0.05 M ammonium acetate buffer pH 5.0. The flow rate was 0.8 mL min^-1^ and the effluent was monitored at 217 nm [40].

For additional metabolite analysis, cultures were prepared as described with 0.5% (v/v) Tween 20 replacing the HMN phase. Both arrangements increase the availability of limonene for the cells. 20 mM acetate was added as co-substrate. To salt out metabolites, 5 g KCO_3_ and 300 μl isopropanol were added to 10 mL culture sampled at the early stationary growth phase. The organic and aqueous phases were separated via centrifugation at 5000 × *g* for 5 min. 1 μL of the upper phase was analyzed for metabolites and their enantiomer-specificity using a gas chromatograph (PerkinElmer Auto System XL; Überlingen, Germany) equipped with a flame ionization detector. Separation was accomplished on a Hydrodex-*β*-6TBDM column (25 m × 0.25 mm; Macherey-Nagel, Düren, Germany) by the following temperature program: injection temperature 200°C; detection temperature 230°C, initial column temperature 80°C for 1 min, increasing to 130°C at a rate of 5°C min^-1^, after 0.5 min further increasing to 230°C at 20°C min^-1^ and stationary for 2 min. For identification of peaks, a 1 μl sample was analyzed on a Trace GC/MS (Thermo Finnigan, Waltham, USA). Separation was performed on a HP-5 column (25 m × 0.2 mm × 0.33 μm; Agilent, Santa Clara, USA) with the following temperature program: injection port temperature 250°C, initial column temperature 60°C for 6 min, increasing to 120°C at 3°C min^-1^ further to 320°C at 40°C min^-1^ and hold for 3 min.

### Genome sequencing and annotation

Genomic DNA was extracted as previously described [41]. The genome of *Castellaniella defragrans* 65Phen was sequenced at the Max Planck Genome Center in Cologne, using the PacBio SMRT system (Pacific Biosciences, Menlo Park, CA). 19858 quality-checked error-corrected reads with at least 4975 bases were *de novo* assembled to a single contig. The average coverage amounted 91 times and around 4 kb overlapped at each end. Open-reading frames were predicted and annotated by the Rapid Annotations using Subsystems Technology (RAST) pipeline [42]. The G+C content was calculated using Artemis [43]. Putative prokaryotic promoters were predicted with BPROM (http://www.softberry.com/berry.phtml?topic=bprom&group=programs&subgroup=gfindb) and putative terminators were identified using WebGeSTer [44]. ANI was calculated according to Goris *et al.*[45]. The complete genome sequence of *Castellaniella defragrans* 65Phen has been deposited at GenBank under the accession number HG916765.

### Label-free quantitative proteome analysis

The soluble and membrane protein enriched proteome of cells grown on acetate and *α*-phellandrene were compared by 2D-SDS-PAGE and LC-ESI-MS/MS. The cyclic monoterpene *α*-phellandrene was used as monoterpene growth substrate because of its origin as enrichment substrate for *Castellaniella defragrans* 65Phen. Anaerobic cultures were grown in a 10 L fermenter with 100 mM nitrate and 10 mM *α*-phellandrene or 50 mM acetate as previously described [23].

For analyzing the soluble protein fraction, cells were disrupted by sonication in lysis buffer (10 mM Tris pH 7.5, 10 mM EDTA pH 8.0) containing 1.7 mM phenylmethanesulfonylfluoride (PMSF) and cell debris was removed by centrifugation. Proteins (80 μg) were separated on 2D SDS-polyacrylamide gels according to their isoelectric point in the pH range of 3 to 10 and to their molecular mass. Proteins were stained with the fluorescence Sypro Ruby protein gel stain (Invitrogen, Darmstadt, Germany). Spots were detected and quantified on gel images using the software Delta2D (Decodon, Greifswald, Germany). Biological triplicates of each condition were fused and overlaid as dual-channel images and individual spot volumes (%Vol) were calculated as proportion of all proteins on the gel images. Spot ratios which represent an at least 2.5-fold change in spot volume, compared to spots of the acetate samples, were considered. All dominant spots and such spots with ratios >2.5 were automatically excised from the gel (Ettan Spo Picker, GE Healthcare), digested with trypsin and spotted onto a matrix-assisted laser desorption/ionization (MALDI)-target (Ettan Spot Handling Workstation, GE Healthcare). High-throughput MALDI-TOF measurements combined with tandem mass spectrometry were performed on a 4800 MALDI-TOF/TOF Analyser (Applied Biosystems, Darmstadt, Germany). Spectra of a mass range from 900 to 3700 Da were detected during the MALDI-TOF analysis. The three strongest peaks were recorded by the MS/MS analysis. With the GPS Explorer Software (Applied Biosystems) Version 3.6, peaks were indexed and assigned to the corresponding amino acid sequences in the *C. defragrans* 65Phen database by the Mascot search engine Version 2.1.04 (Matrix Science Ltd, Boston, MA, USA). Proteins of at least 25% sequence coverage, minimum 2 unique assigned peptides and a Mowse score value of 75 or higher were treated as identified.

To analyze the membrane protein-enriched fraction, cells were disrupted with lysis buffer (50 mM Tris pH 7.5, 1 mM PMSF) by sonication. Cell debris was removed by short centrifugation and membranes were pelleted by additional ultracentrifugation (100,000 × *g*, 1 h, 4°C). Following the protocol established by Eymann *et al*. [46], the protein pellet was homogenized and washed in high-salt buffer (20 mM Tris pH 7.5, 1 M NaCl), alkaline buffer (0.1 M Na_2_CO_3_-HCl pH 11, 0.1 M NaCl) and 50 mM triethylammonium bicarbonate (TEAB) buffer (pH 7.8) with ultracentrifugation (100,000 × *g*, 1 h, 4°C) after each washing step. The final pellet was resolved in 50 mM TEAB buffer. Samples of 15 μg protein were separated by 1D SDS-PAGE in two technical replicates per condition. After staining with Coomassie Brilliant Blue, each gel lane was divided into 10 slices which were excised and individually analyzed. Proteins were in gel-digested with trypsin, separated by reverse phase chromatography using a nano-Acquity UPLC System (Waters, Milford, MA, USA) and analyzed by MS/MS in a LTQ-Orbitrap mass spectrometer (Thermo Fisher Scientific, Waltham, MA, USA). Spectra were assigned to the corresponding amino acid sequence of the *C. defragrans* 65Phen database using Sorcerer-SEQUEST (SEQUEST version 2.7 revision 11, Thermo Scientific) including Scaffold 3_00_08 (Proteome Software Inc., Portland, OR, USA). SEQUEST was searched with a parent ion tolerance of 10 ppm and a fragment ion mass tolerance of 1.00 Da. For protein identification, a stringent SEQUEST filter for peptides was used (Xcorr versus charge state: 1.80 for singly, 2.2 for doubly, and 3.3 for triply charged ions and deltaCn value greater than 0.10) and at least two unique peptides per proteins were required for identification. Protein fractions from both conditions were considered to be different if no peptides were detected in the alternative fraction or if a significant difference was shown by the Scaffold internal *t*-test analysis (threshold of 95%).

For continuity of the protein abbreviations, GeDH and GaDH [26] were renamed to GeoA and GeoB according to their gene abbreviations.

### Deletion mutagenesis

An in frame deletion mutant of *geoB* was created as described for the *ldi* gene [27]. The 5'-flanking region (2015 bases) was amplified with the primers GeoB1_XbaI_F (*tctaga*agagatcgtgaccagctttcc) and GeoB2_NdeI_R (*catatg*catcgagggtgtctcctgagt) and the 3'-flanking region (1967 bases) with GeoB3_NdeI_F (*catatg*taggatggacggacaccagg) and GeoB4_HindIII_R (*aagctt*gatgccgacggcgaacttg). Both amplicons were ligated in a pK19mobsacB plasmid via subcloning using a pCR4-TOPO vector (Invitrogen, Darmstadt, Germany). The constructed plasmid pK19mobsacB*ΔgeoB* was transferred in *C. defragrans* 65Phen by conjugation and colonies were screened for a second recombination event.

### Transposon insertion mutagenesis

Transposon insertion mutants of *C. defragran*s 65Phen were created by biparental conjugation. 2 mL of overnight cultures of *C. defragrans* 65Phen anaerobically grown on 20 mM acetate and 20 mM nitrate with 150 μg mL^-1^ rifampicin and of *E.coli* W20767 (lysogeny broth medium with 50 μg mL^-1^ kanamycin) carrying the plasmid pRL27 were spin down at 8000 × *g* for 5 min. The pellets were washed twice and resuspended in 100 μL mineral medium. The optical density at 600 nm of both cell suspensions was adjusted to 1, combined equally (100 μL) and added as one drop on a mineral medium plate (1.5% agar) containing 50 mM acetate without antibiotics. After 24 h incubation at 28°C, cells were resuspended from the plate with 1 mL mineral medium. 100 μL of cell suspension were plated in different dilutions on mineral medium agar plates containing 50 mM acetate, 25 μg mL^-1^ kanamycin and 150 μg mL^-1^ rifampicin. The plates were anaerobically incubated in a jar for 4 days at 28°C. Mutants affected in the limonene metabolism were identified by replica plating (replicator stamp, Carl Roth GmbH + Co. KG, Karlsruhe, Germany). The replicon was incubated in an anaerobic jar with a limonene-enriched headspace for at least two days. Putative mutant colonies were transferred twice to new plates with acetate or limonene as carbon source to confirm the phenotype and the insertion position was determined by a direct sequencing approach. The genomic DNA was isolated from a liquid culture using FastDNA Spin Kit for Soil (MP Biomedicals, OH, USA). 2 to 3 μg genomic DNA were applied for the sequencing PCR reaction using BigDye Terminator v3.1 Cycle Sequencing Kit (Applied Biosystems, Life Technologies Corporation, Carlsbad, CA, USA). The primers tpnRL 17-1 and tpnRL 13-2 [31] were used with following program: 95°C for 5 min, 100 cycles of 96°C for 30 sec, 52°C for 20 sec and 60°C for 4 min. The fragments were sequenced with an ABI Prism 3130*xl* Genetic Analyzer (Applied Biosystems Life Technologies Corporation, Carlsbad, CA, USA).

## Competing interests

The authors declare that they have no competing interests.

## Authors’ contributions

JH and JP planed the study. EMD performed the transposon insertion mutagenesis. SM, DB and RS supported the proteome analysis. BH and RR sequenced the genome of *Castellaniella defragrans* 65Phen. JP analyzed the genome and proteome, created the deletion mutant of *geoB,* did physiological experiments and metabolite identification. JP and JH analyzed the data and wrote the manuscript. All authors read and approved the final manuscript.

## Supplementary Material

Additional file 1: Table S1 Up-regulated proteins of cells grown with *α*-phellandrene. ^a^2D, identified with 2D-SDS-PAGE and MALDI-TOF-MS; LC, identified with LC-ESI-MS/MS. ^b^++ peptides only identified in α-phellandrene fraction, + increase in α-phellandrene fraction, 0 ratio remained unchanged. Click here for file

Additional file 2: Figure S1 Growth of *C. defragrans* 65Phen transposon mutants. (●) *C. defragrans* 65Phen wild type, (▽) *ctmA*::Tn5a, (■) *ctmA*::Tn5b, (◊) *ctmB*::Tn5a, (▲) *ctmB*:Tn5b and (○) *ctmE*::Tn5 (for details see Tabl. 3) in anoxic incubations with 3 mM limonene (A) and 3 mM perillyl alcohol (B) as well as oxic incubations with 3 mM limonene (C) and 3 mM perillyl alcohol (D) are represented. Click here for file
