# Supplementary material for: The oxygen-independent metabolism of cyclic monoterpenes in Castellaniella defragrans 65Phen
Source: BMC Microbiol. 2014 Jun 21;14:164. doi: 10.1186/1471-2180-14-164 (PMC4109377; doi:10.1186/1471-2180-14-164)
Supplement: Additional file 1: Table S1 — Up-regulated proteins of cells grown with α-phellandrene. a2D, identified with 2D-SDS-PAGE and MALDI-TOF-MS; LC, identified with LC-ESI-MS/MS. b++ peptides only identified in α-phellandrene fraction, + increase in α-phellandrene fraction, 0 ratio remained unchanged. [file 1471-2180-14-164-S1.pdf]

Protein_id	2D/LC[a]	2D fold change / LC enrichment level[b]	No. of matching unique peptides	% Coverage	Gene annotation	Related gene product E value	% Identity	Organism	Accession no.
CDM22555	LC	++	2	6	histidinol dehydrogenase	0.0	78	*Alcaligenes faecalis*	WP_003805416
CDM22588	LC	+	27	33	short shain acyl-CoA dehydrogenase	0.0	70	*Achromobacter arsenitoxydans*	WP_008168415
CDM22589	LC	++	4	14	3-ketoacyl-CoA thiolase	0.0	75	*Pusillimonas* sp. T7-7	YP_004418047
CDM22590	LC	++	6	9	bifunctional enoyl-CoA hydratase / 3-hydroxyacyl-CoA dehydrogenase	0.0	66	*Pusillimonas* sp. T7-7	YP_004418048
CDM22592	LC	++	5	18	long-chain fatty acid transport protein	0.0	58	*Alcaligenes* sp. HPC1271	WP_009462456
CDM22790	2D/LC	5.0/+	3/2	31/21	hypothetical protein	4E-37	63	*Pusillimonas noertemannii*	WP_017525672
CDM22815	2D	7.2	28	55	ABC-type dipeptide transport system, periplasmic	0.0	62	*Acidovorax* sp. CF316	WP_007849611
CDM22834	LC	+	7	44	ubiquinol-cytochrome C reductase iron-sulfur subunit	2E-126	79	*Pusillimonas* sp. T7-7	YP_004418271
CDM22981	LC	++	3	10	hypothetical protein	0.0	87	*Dechloromonas aromatica* RCB	YP_283598
CDM22999	LC	++	3	8	hypothetical protein	0.0	63	*Pusillimonas noertemannii*	WP_017525303
CDM23107	2D	4.9	20	54	ABC transport protein, substrate-binding component	0.0	83	*Pusillimonas* sp. T7-7	YP_004417743
CDM23163	LC	++	6	21	threonine dehydratase	0.0	83	*Pusillimonas* sp. T7-7	YP_004418520
CDM23168	LC	++	3	14	hypothetical protein	5E-58	52	*Pusillimonas noertemannii*	WP_017524873
CDM23355	LC	++	2	10	carbamoyl phosphate synthase small subunit	0.0	81	*Pusillimonas* sp. T7-7	YP_004415263
CDM23364	LC	++	2	6	phosphoglucosamine mutase	0.0	76	*Pusillimonas* sp. T7-7	YP_004415277
CDM23503	2D	4.0	10	57	ketohydroxyglutarate aldolase	1E-98	73	*Pusillimonas* sp. T7-7	YP_004415557
CDM23555	LC	+	6	16	3-oxoacyl-ACP synthase	0.0	87	*Pusillimonas* sp. T7-7	YP_004416855
CDM23571	2D/LC	10.6/++	12/2	38/7	ABC transport protein, substrate-binding component	0.0	81	*Pusillimonas* sp. T7-7	YP_004416836
CDM23572	2D/LC	3.2/+	20/5	26/12	methylmalonate-semialdehyde dehydrogenase	0.0	80	*Bordetella bronchiseptica* 253	YP_006970713
CDM23583	2D/LC	3.4/0	19/6	35/11	acetyl-CoA synthetase	0.0	80	*Pusillimonas* sp. T7-7	YP_004416829
CDM23629	LC	+	11	34	ABC-type proline/glycine betaine transport system, ATPase component	0.0	81	*Pusillimonas* sp. T7-7	YP_004416797
CDM23645	2D	3.0	15	44	ABC transport protein, substrate-binding component	0.0	74	*Pusillimonas* sp. T7-7	YP_004416670
CDM23900	LC	++	4	12	histidinol-phosphate aminotransferase	0.0	73	*Bordetella petrii* DSM 12804	YP_001630494
CDM23916	2D/LC	4.8/++	10/3	61/17	alkyl hydroperoxide reductase subunit C-like protein	5E-129	84	*Pusillimonas* sp. T7-7	YP_004416636
CDM23981	LC	++	3	23	DNA-3-methyladenine glycosidase I	6E-87	66	*Pusillimonas* sp. T7-7	YP_004418129
CDM24003	LC	++	3	10	xanthine permease	0.0	61	*Pusillimonas* sp. T7-7	YP_004418527
CDM24020	LC	++	2	7	coproporphyrinogen III oxidase, oxygen-independent	0.0	68	*Pusillimonas* sp. T7-7	YP_004415790
CDM24134	LC	++	3	11	4-hydroxy-3-methylbut-2-en-1-yl diphosphate synthase	0.0	86	*Bordetella bronchiseptica* 253	YP_006967058
CDM24151	LC	+	7	19	aldehyde dehydrogenase, NAD-dependent	0.0	84	*Pusillimonas* sp. T7-7	YP_004417620
CDM24267	LC	++	2	14	transcriptional regulator, MerR family	1E-68	83	*Pusillimonas* sp. T7-7	YP_004416423
CDM24360	LC	++	4	5	5-methyltetrahydropteroyltriglutamate / homocysteine S-methyltransferase	0.0	77	*Bordetella petrii* DSM 12804	YP_001630668
CDM24415	2D	2.6	7	43	nitroreductase	3E-69	58	*Pusillimonas* sp. T7-7	YP_004416172

CDM24438	LC	++	2	9	uridylate kinase	6E-145	89	*Pusillimonas noertemannii*	WP_017523417
CDM24452	LC	++	5	22	PEP synthetase regulatory protein	8E-151	78	*Pusillimonas* sp. T7-7	YP_004416206
CDM24483	LC	++	2	11	chemotaxis protein MotA	7E-128	70	*Alcaligenes* sp. EGD-AK7	ERI34412
CDM24494	LC	++	4	22	transcriptional regulator	2E-137	89	*Pusillimonas* sp. T7-7	YP_004416261
CDM24535	2D/LC	6.2/++	18/3	49/10	ABC-type glycerin-3-phosphate transport system, substrate-binding component	0.0	76	*Pusillimonas* sp. T7-7	YP_004416342
CDM24582	LC	++	2	16	glutathione S-transferase	2E-127	83	*Nitrosomonas eutropha* C91	YP_747060
CDM24665	LC	++	2	8	hypothetical protein	6E-128	62	*Pusillimonas* sp. T7-7	YP_004416004
CDM24668	LC	++	4	13	branched-chain alpha-keto acid dehydrogenase E1 component	0.0	84	*Pusillimonas* sp. T7-7	YP_004416001
CDM24732	LC	++	4	15	bifunctional riboflavin kinase/FMN adenylyltransferase	6E-162	71	*Pusillimonas noertemannii*	WP_017523660
CDM24744	2D	3.0	3	47	peptidyl-prolyl cis-trans isomerase	1E-48	84	*Pusillimonas* sp. T7-7	YP_004417531
CDM24755	2D	3.1	4	25	superoxide dismutase [Fe]	9E-117	82	*Bordetella bronchiseptica* 253	YP_006967574
CDM24805	LC	++	2	9	flagellar biosynthesis protein FliH	1E-58	46	*Bordetella pertussis* Tohama I	NP_880144
CDM24812	LC	++	3	30	flagellar biosynthesis protein FliO	2E-23	59	*Pusillimonas noertemannii*	WP_017525059
CDM24890	LC	++	7	49	hns-like transcription regulator protein	3E-43	59	*Pusillimonas noertemannii*	WP_017525369
CDM24905	LC	++	3	9	hypothetical protein	0.0	81	*Oligotropha carboxidovorans* OM5	YP_002288631
CDM24945	LC	++	2	8	ornithine cyclodeaminase	5E-138	59	*Cupriavidus taiwanensis*	WP_018007161
CDM24950	2D	2.8	8	40	hypothetical protein	2E-49	60	*Bordetella bronchiseptica* RB50	NP_888469
CDM24952	LC	++	2	7	phosphate starvation protein PhoH	0.0	82	*Pusillimonas noertemannii*	WP_017524275
CDM25009	2D/LC	3.4/+	13/6	69/34	acetoacetyl-CoA reductase	1E-159	91	*Pusillimonas* sp. T7-7	YP_004415605
CDM25024	LC	++	3	8	bifunctional enoyl-CoA hydratase/phosphate acetyltransferase	0.0	72	*Alicycliphilus denitrificans* BC	YP_004128659
CDM25241	LC	++	6	20	acyl-CoA dehydrogenase	0.0	82	*Thauera terpenica* 58Eu	EPZ16240
CDM25246	2D/LC	3.0/++	9/2	38/11	3-hydroxyacyl-CoA dehydrogenase	5E-167	82	*Azoarcus* sp. KH32C	YP_007598290
CDM25250	LC	++	5	10	perillyl-CoA hydratase	0.0	58	*Thauera terpenica* 58Eu	EPZ16258
CDM25251	2D/LC	18/++	13/4	64/20	4-isopropenyl-2-oxo-cyclohexane-1-carboxyl-CoA hydrolase	4E-167	88	*Azoarcus* sp. KH32C	YP_007598294
CDM25252	2D/LC	3.0/++	10/2	51/7	2-hydroxy-4-isopropenyl-cyclohexane-1-carboxyl-CoA dehydrogenase	1E-153	85	*Azoarcus* sp. KH32C	YP_007598295
CDM25254	LC	++	5	17	acyl-CoA dehydrogenase	0.0	90	*Azoarcus toluclasticus*	WP_018990727
CDM25255	LC	++	14	46	oxidoreductase, FAD-binding	0.0	73	*Azoarcus toluclasticus*	WP_018990723
CDM25256	2D/LC	19/++	4/2	35/14	(R)-specific enoyl-CoA hydratase	1E-88	85	*Thauera terpenica* 58Eu	EPZ15051
CDM25258	LC	++	3	8	acyl-CoA dehydrogenase	0.0	92	*Thauera terpenica* 58Eu	EPZ15053
CDM25259	LC	++	16	32	RND efflux transporter, outer membrane component	0.0	62	*Thauera terpenica* 58Eu	EPZ15054
CDM25260	LC	++	4	27	RND efflux transporter, periplasmic component	0.0	80	*Thauera terpenica* 58Eu	EPZ15055
CDM25261	LC	++	5	21	RND efflux transporter, periplasmic component	4E-142	68	*Thauera terpenica* 58Eu	EPZ15056
CDM25262	LC	++	9	17	RND efflux transporter, inner membrane component	0.0	81	*Thauera terpenica* 58Eu	EPZ15057
CDM25263	LC	++	2	4	acetoacetyl-CoA synthetase	0.0	84	*Thauera terpenica* 58Eu	EPZ15058
CDM25265, GeoC	LC	++	4	16	perillate--CoA ligase	0.0	71	*Thauera terpenica* 58Eu	EPZ15060

CDM25267, GeoA	2D/LC	42/++	15/4	52/19	geraniol dehydrogenase	0.0	84	*Thauera terpenica* 58Eu	EPZ14350
CDM25268	LC	++	2	9	hypothetical protein	8E-117	74	*Thauera terpenica* 58Eu	EPZ14349
CDM25272, Ldi	LC	++	2	8	linalool dehydratase-isomerase precursor	8E-14	25	*Stereum hirsutum* FP-91666 SS1	EIM80109
CDM25275	LC	++	4	5	acyl-CoA dehydrogenase	4E-106	54	*Azoarcus toluclasticus*	WP_018990670
CDM25279	LC	++	4	8	hypothetical protein	1E-120	68	*Pseudomonas* sp. 19-rlim	AEO27372
CDM25281, GeoB	2D/LC	15/++	16/14	43/37	geranial dehydrogenase	0.0	91	*Thauera terpenica* 58Eu	EPZ16290
CDM25282	LC	++	3	8	acyl-CoA dehydrogenase	0.0	89	*Thauera terpenica* 58Eu	EPZ16289
CDM25284, CtmG	LC	++	3	13	hypothetical protein	5E-35	39	*Azoarcus* sp. KH32C	YP_007598506
CDM25285, CtmF	LC	++	14/2	35/10	NADH:ferredoxin oxidoreductase	4E-147	56	*Caulobacter* sp.AP07	WP_007674692
CDM25286, CtmE	2D/LC	6.3/++	3	36	ferredoxin, 2Fe-2S	1E-32	50	*Caulobacter crescentus* CB15	NP_422318
CDM25289, CtmB	2D/LC	3.4/++	17/9	40/27	limonene dehydrogenase, beta subunit	4E-131	41	*Deltaproteobacterium* NaphS2	WP_006422074
CDM25290, CtmA	LC	++	5	11	limonene dehydrogenase, alpha subunit	5E-57	30	*Deltaproteobacterium* NaphS2	WP_006422074
CDM25291	LC	++	3	11	acetyl-CoA acetyltransferases	0.0	79	*Thauera terpenica* 58Eu	EPZ16237
CDM25294	LC	++	3	10	hypothetical protein	2E-83	68	*Thauera terpenica* 58Eu	EPZ16282
CDM25298	LC	++	4	10	hypothetical protein	0.0	72	*Thauera terpenica* 58Eu	EPZ16231
CDM25299	LC	++	4	13	acetyl-CoA acetyltransferase	0.0	90	*Thauera terpenica* 58Eu	EPZ16230
CDM25301	LC	++	5	22	electron transfer flavoprotein, beta subunit	7E-133	78	*Thauera terpenica* 58Eu	EPZ16226
CDM25303	LC	++	3	14	isobutyryl-CoA dehydrogenase	0.0	83	*Pusillimonas* sp. T7-7	YP_004416217
CDM25304	LC	++	3	11	3-hydroxyisobutyryl-CoA hydrolase	7E-166	63	*Pusillimonas* sp. T7-7	YP_004416218
CDM25305	2D	4.9	9	40	3-hydroxyisobutyrate dehydrogenase	4E-157	80	*Achromobacter arsenitoxydans*	WP_008167752
CDM25315	LC	++	2	16	NUDIX hydrolase	7E-91	66	*Pusillimonas noertemannii*	WP_017525801
CDM25396	LC	++	6	16	benzoyl-CoA oxygenase component B	0.0	85	*Pusillimonas noertemannii*	WP_017525211
CDM25490	LC	++	3	21	deoxycytidine triphosphate deaminase	2E-128	96	*Alcaligenes faecalis*	WP_003803918
CDM25598	LC	++	3	5	SAM-dependent methyltransferase	0.0	70	*Cellvibrio japonicus* Ueda107	YP_001982373
CDM25622	LC	++	5	8	GTP-binding protein	0.0	52	*Thauera* sp. MZ1T	YP_002354075
CDM25656	LC	++	6	13	hypothetical protein	0.0	55	*Gloeobacter violaceus* PCC 742	NP_925494
CDM25709	LC	++	5	13	phosphoglucomutase	0.0	72	*Pusillimonas* sp. T7-7	YP_004417296
CDM25726	LC	++	3	16	UDP-glucose dehydrogenase	0.0	85	*Bordetella avium* 197N	YP_785044
CDM25743	LC	+	11	23	glycyl-tRNA synthetase beta subunit	0.0	70	*Bordetella petrii* DSM 12804	YP_001629381
CDM25779	LC	++	4	17	UDP-N-acetylmuramate--L-alanine ligase	0.0	85	*Pusillimonas* sp. T7-7	YP_004415177
CDM25780	LC	++	2	8	UDP-N-acetylglucosamine--N-acetylmuramyl-(pentapeptide) pyrophosphoryl-UDP N-acetylglucosamine transferase	4E-169	71	*Pusillimonas noertemannii*	WP_017524749
CDM25847	LC	++	3	15	3-methyladenine DNA glycosylase	5E-120	75	*Alicycliphilus denitrificans* BC	YP_004125978
CDM25890	LC	++	4	11	ubiquinone biosynthesis hydroxylase UbiH	8E-151	60	*Pusillimonas noertemannii*	WP_017525509
CDM25903	2D/LC	4.4/0	9/3	36/17	F0F1 ATP synthase subunit gamma	8E-177	83	*Pusillimonas* sp. T7-7	YP_004417598
CDM25967	2D	3.5	22	63	PmbA peptidase	0.0	74	*Pusillimonas* sp. T7-7	YP_004418694
CDM26011	LC	++	2	7	ABC-type amino acid transport system, permease component	0.0	81	*Pusillimonas sp.* T7-7	YP_004417548

CDM26059	2D/LC	-1.1/++	9/5	16/12	outer membrane esterase	0.0	52	*Pseudogulbenkiania ferrooxidans*	WP_021475494
CDM26067	2D/LC	3.3/++	14/2	54/5	ABC-type dipeptide transport system, periplasmic component	0.0	62	*Enterobacter aerogenes* KCTC 2190	YP_004590478
