# Supplementary figures and images for: The oxygen-independent metabolism of cyclic monoterpenes in Castellaniella defragrans 65Phen
Source: BMC Microbiol. 2014 Jun 21;14:164. doi: 10.1186/1471-2180-14-164 (PMC4109377; doi:10.1186/1471-2180-14-164)

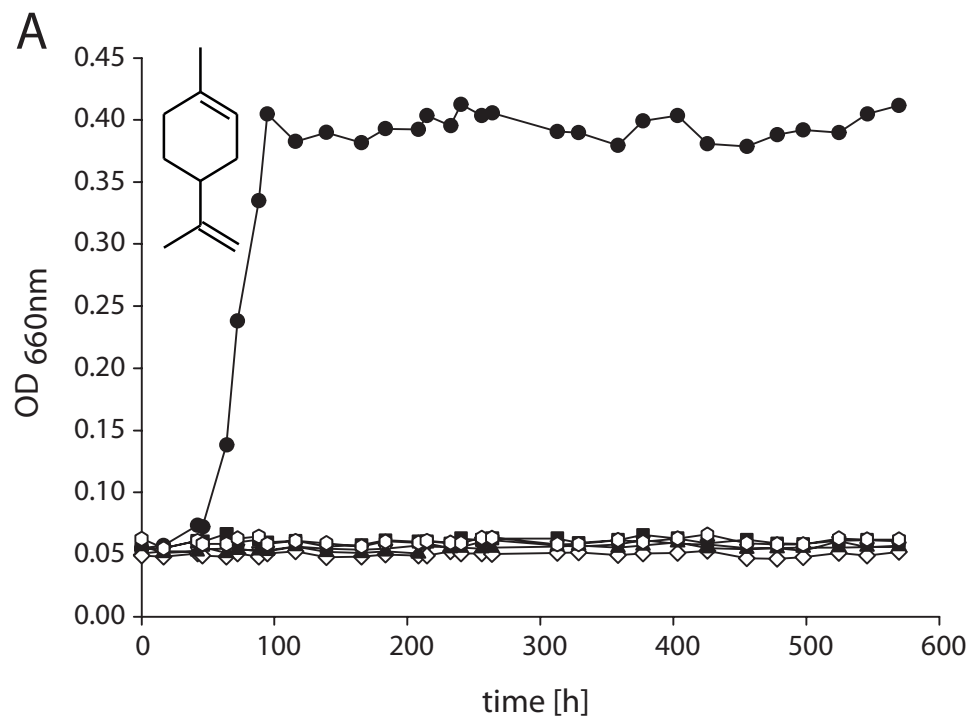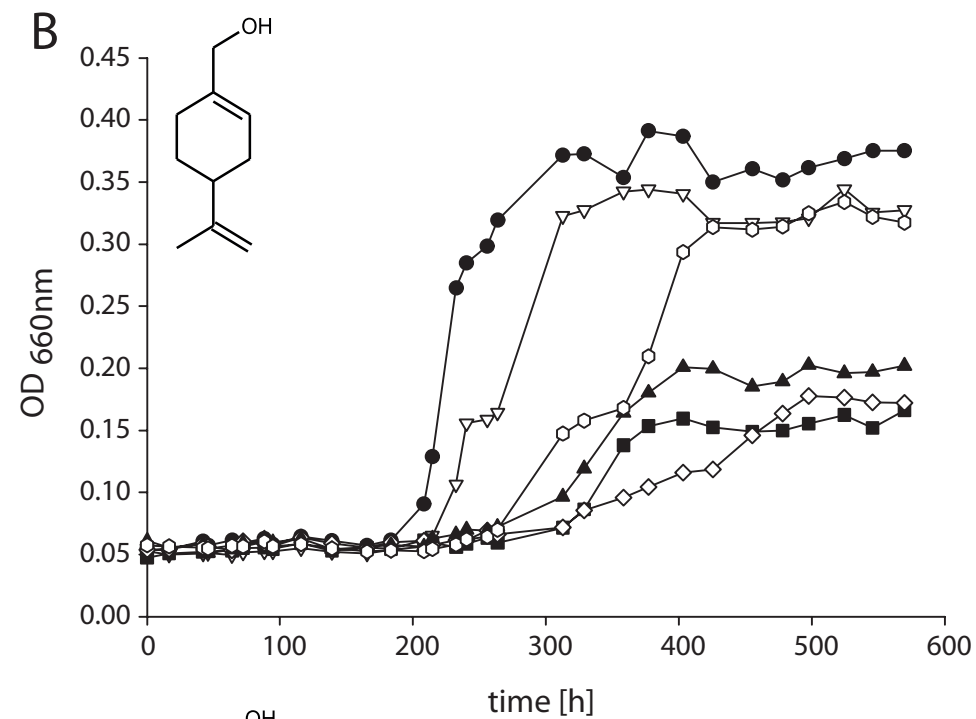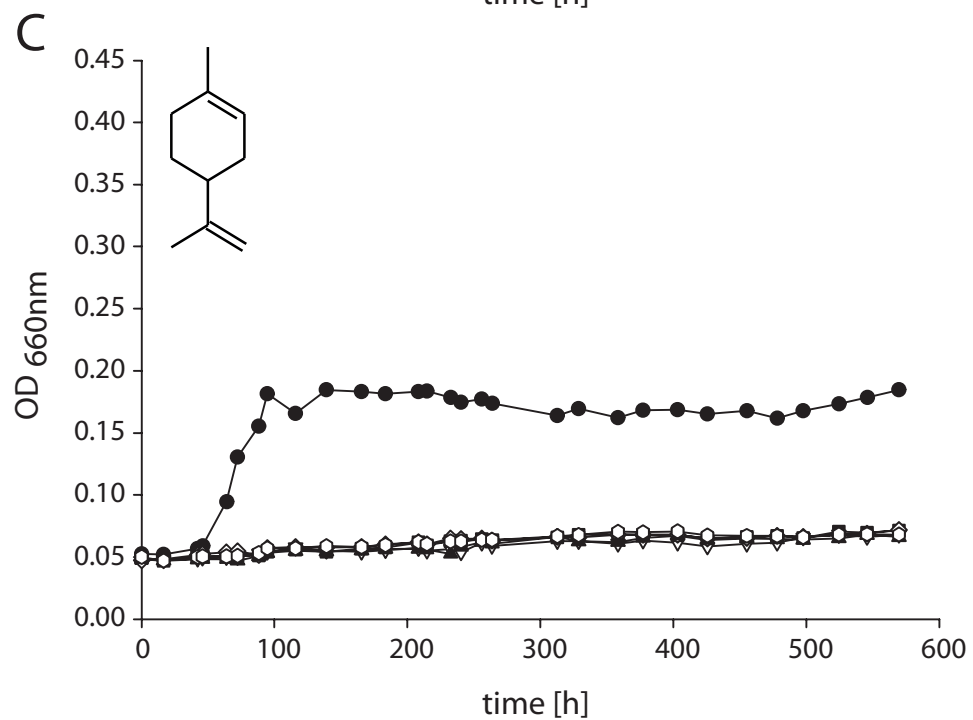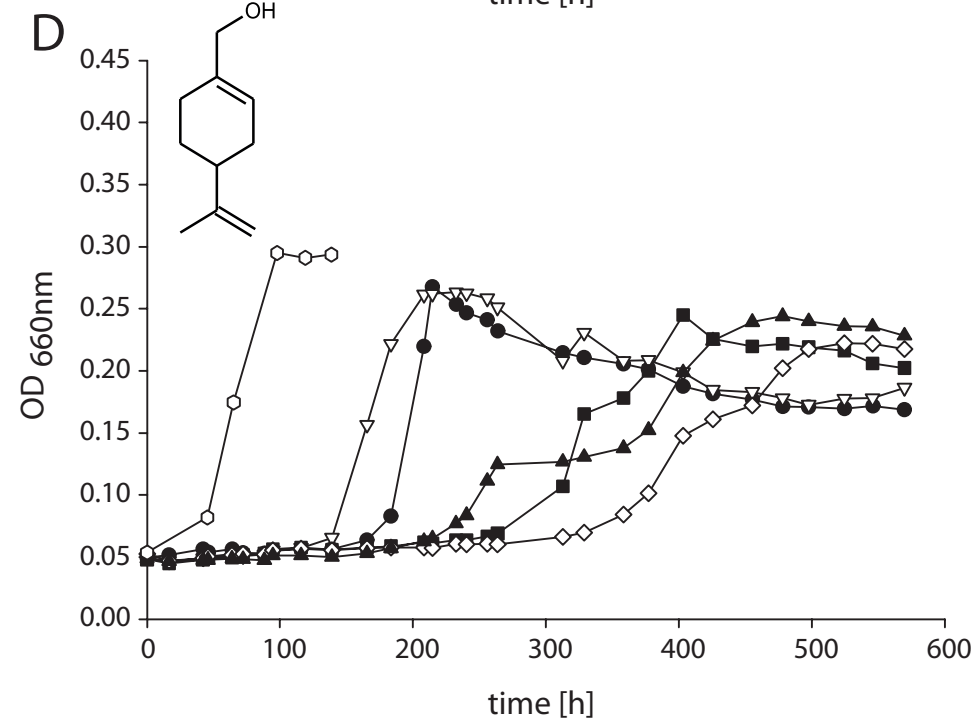

Supplement: Additional file 2: Figure S1 — Growth of C. defragrans 65Phen transposon mutants. (●) C. defragrans 65Phen wild type, (▽) ctmA::Tn5a, (■) ctmA::Tn5b, (◊) ctmB::Tn5a, (▲) ctmB:Tn5b and (○) ctmE::Tn5 (for details see Tabl. 3) in anoxic incubations with 3 mM limonene (A) and 3 mM perillyl alcohol (B) as well as oxic incubations with 3 mM limonene (C) and 3 mM perillyl alcohol (D) are represented. [file 1471-2180-14-164-S2.pdf]
